# A novel *Bacteroides* metallo-β-lactamase (MBL) and its gene (*crxA*) in *Bacteroides xylanisolvens* revealed by genomic sequencing and functional analysis

**DOI:** 10.1093/jac/dkac088

**Published:** 2022-03-17

**Authors:** József Sóki, Uwe Lang, Ulrike Schumacher, István Nagy, Ágnes Berényi, Tamás Fehér, Katalin Burián, Elisabeth Nagy

**Affiliations:** 1 Department of Medical Microbiology, Albert Szent-Györgyi Health Center and Albert Szent-Györgyi Medical School, University of Szeged, Szeged, Hungary; 2 Medical Care Centre Laboratory Münster, Münster, Germany; 3 Medical Care Centre Laboratory Ravensburg, Ravensburg, Germany; 4 Institute of Biochemistry, Biological Research Centre, Loránd Eötvös Research Network, Szeged, Hungary; 5 SEQOMICS Ltd, Mórahalom, Hungary

## Abstract

**Objectives:**

We sought to characterize the carbapenem resistance mechanism of *Bacteroides xylanisolvens* 14880, an imipenem-resistant strain from Germany, and assess its prevalence.

**Methods:**

Antimicrobial susceptibilities were determined using agar dilution or Etest methodology and specific imipenemase activity was detected. The genomic sequence of *B. xylanisolvens* 14880 was determined and analysed for antibiotic resistance genes and genomic islands. We also used gene transfer to a carbapenem susceptible host, along with 5′-RACE, conventional PCR with capillary sequencing and RT–PCR-based screening.

**Results:**

*B. xylanisolvens* 14880 displayed resistance to carbapenems and produced high specific imipenemase activity. Its genomic sequence was 6.1 Mbp and a class B1 β-lactamase gene (termed *crxA*) was detected in it. *crxA* was carried on a putative genomic island with insertion sequence (IS) elements and a putative GNAT (Gcn5-like acetyltransferase) toxin gene. Promoter localization by 5′-RACE and gene targeting to an imipenem-susceptible *Bacteroides* host indicated that it is activated by an IS*1380*-like IS element and it can confer carbapenem resistance. The PCR screening of *Bacteroides* strains showed that *crxA* was specific to *B. xylanisolvens* with a carriage rate of 16.7%.

**Conclusions:**

*B. xylanisolvens* strains can harbour a carbapenem resistance gene, which has many similarities to the ‘*cfiA* system’: metallo-β-lactamase (MBL), IS element activation, carriage of a GNAT toxin gene, specific for a unique *Bacteroides* species with a significant prevalence.

## Introduction

The most effective antibiotics that target anaerobic *Bacteroides* infections are carbapenems, metronidazole, β-lactam/β-lactamase combinations and tigecycline.^1^ The carbapenem resistance of *Bacteroides fragilis* is mediated by the CfiA metallo-β-lactamase (MBL), which is activated by the integration of insertion sequence (IS) elements upstream of the resistance gene.^2^ The *cfiA* gene is contained on a DNA segment (‘*cfiA* element’) that is specific for division II strains and it is always accompanied by two ORFs encoding acetylases (GNAT – Gcn5-like acetyltransferase and XAT/*vat* – xenobiotic acetylase, which are now thought to form a toxin–antitoxin pair).^3–5^ The CfiA-mediated resistance mechanism can limit the treatment of *B. fragilis* infections, but, so far, there have been no major concerns regarding non-*fragilis Bacteroides* species in terms of carbapenem resistance, despite its detection; in the USA it has been reported only at a very low prevalence (usually between 0% and 0.5% for different species).^6^ Additionally, except for a few cases, namely a *Parabacteroides distasonis* from the USA,^7^ two other *Bacteroides* strains isolated from faeces in our laboratory^8^ and a *Bacteroides thetaiotaomicron* strain also from the USA,^9^ no carbapenem-resistant, non-*fragilis Bacteroides* strain has been characterized in more detail.

*Bacteroides xylanisolvens* was described in 2008 based on the xylanolytic activity of some *Bacteroides* strains isolated from human faecal samples.^10^ It is very closely related to *Bacteroides ovatus* so that it is scarcely distinguishable from it by MALDI-TOF MS or 16S rDNA sequencing, but some phenotypic tests may be useful for this purpose.^11^

In this study we report the isolation of a highly imipenem-resistant *B. xylanisolvens* strain, its phenotypic and molecular characterization and the description of its novel MBL gene and its prevalence.

## Materials and methods

### Bacterial strains and cultivation

*B. xylanisolvens* 18440 (Table 1) was isolated in 2016 from one of the microbiological samples of a patient diagnosed as having thoracic empyema as the main symptom and posterior wall infarction, which was resolved with bypass-operation surgery (2015). The therapy for the pleural empyema in 2016 included partial pleurectomy, drainage, Kerlix rolls and antibiotic treatment (piperacillin/tazobactam IV 3 × 4.5 g and after its insufficiency imipenem IV 3 × 1 g and later 4 × 0.5 g). Following these treatments and an 11 week hospital stay the patient’s state improved and he returned home with regular wound observation and care.

**Table 1.** List of strains and the results of examinations of the prevalence and activation mechanisms of *crxA* genes in *Bacteroides* strains

Study/strains			Imipenem MIC (mg/L)	*crxA*	IS*1380*-like IS	*crxA* upstream region^a^
Original identification	identification using MALDI-TOF MS	identification using *rpoB* sequencing				
*B. xylanisolvens* 14880	*B. xylanisolvens*	*B. xylanisolvens*	128	+	+	+ (1.8 kb)
Study 1^8^						
* B. fragilis* 3035	*B. fragilis*	NA	0.25 (4)^b^	−	NT	NA
* Bacteroides capillosus* 427/1	*Pseudoflavonifractor capillosus*	NA	2 (32)^b^	−	NT	NA
* Bacteroides distasonis* 22/1	*B. ovatus/xylanisolvens*	*B. xylanisolvens*	32	+	−	− (208 bp)
* Bacteroides vulgatus* 5/4	*B. vulgatus/dorei*	NA	2 (32)^b^	−	NT	NA
Study 2^12^ (400 *Bacteroides* and *Parabacteroides* isolates)						
* B. ovatus/xylanisolvens* (*n *= 21)	*B. ovatus/xylanisolvens* (*n *= 3)	*B. ovatus* (*n *= 3)	NT	−	NT	NA
	*B. xylanisolvens* (*n *= 15)	*B. xylanisolvens* (*n *= 15)	NT	−	NT	NA
	*B. xylanisolvens* D85	*B. xylanisolvens*	0.5 (8)^b^	+	−	− (208 bp)
	*B. xylanisolvens* Sz9	*B. xylanisolvens*	0.125 (0.5)^b^	+	−	− (208 bp)
	*B. xylanisolvens* P8	*B. xylanisolvens*	2	+	+ (PCR mapping −)^c^	− (208 bp)

NA, not applicable; NT, not tested.

a Sizes of the amplified upstream fragments of the *crxA* genes are shown in parentheses.

b Heterogeneous resistance phenotype; full inhibition zone is indicated first then the value where individual resistant colonies disappear is noted in parentheses.

c The strain contains an IS*1380*-like IS, but it could not be mapped to the *crxA* gene.

Four hundred and four other *Bacteroides* strains were also included from two previous studies (see Table 1); one that screened imipenem-resistant *Bacteroides* strains from faeces in Hungary and the UK (4 strains, Study 1) and one that was a *Bacteroides* antibiotic resistance survey in Hungary in 2014–16 (400 strains, Study 2).^8,12^ The methods of bacterial cultivations and manipulations have been described previously.^5^

### Antimicrobial susceptibility tests and carbapenemase assay

Antimicrobial susceptibilities were determined using Etest methodology, as recommended by the supplier (bioMérieux), or agar dilution in the case of the 400 clinical *Bacteroides* and *Parabacteroides* strains (Study 2; Table 1). Carbapenemase production by *B. xylanisolvens* 14880 was measured as described previously.^8^

### WGS and bioinformatic analyses

WGS of *B. xylanisolvens* 14880 was performed using Illumina mate-paired sequencing technology, the resulting sequence was submitted to GenBank (www.ncbi.nlm.nih.gov, acc. no. PRJNA481227) and the WGS data were further analysed using ResFinder and IslandViewer 4 for the presence of antibiotic resistance genes (80% coverage and 30% homology parameters) and genomic islands, respectively.^13,14^

### Determination of the promoter of crxA in B. xylanisolvens 14880

5′-RACE was used to determine the transcription initiation site of the *crxA* gene in *B. xylanisolvens* 14880 using a kit (Roche) (using steps as recommended by the supplier). Template total RNA was isolated using the HighPure RNA Isolation Kit (Roche) and the contaminating DNA was digested using DNase I (Thermo Fisher Scientific). The sequences of the gene-specific primers are given in Table S1 (available as Supplementary data at *JAC* Online) with the corresponding annealing temperatures used in the PCR steps in 50 μL final volumes using the DreamTaq endpoint PCR Master Mix (Thermo Fisher Scientific). The RACE PCR fragment was inserted into the pJET2.1 vector (Thermo Fisher Scientific) and the resulting constructs were sequenced using Sanger capillary sequencing (Life Technologies).

### Gene transfer and PCR experiments

The *crxA* gene was amplified by PCR using different lengths of upstream regions (Table 2 and Figure S1), digested using BamHI and ligated into the BamHI site of the *Bacteroides-Escherichia coli* shuttle vector pFD288.^15^ The ligation mixtures were transformed by electroporation into the *E. coli* DH5α strain and the construct was transferred into the *B. fragilis* 638R host using triparental mating.^15^

**Table 2.** Carbapenem MICs of the upstream constructs of *crxA* from *B. xylanisolvens* 14880 in *B. fragilis* 638R^a^

Carbapenem	MIC (mg/L)				
	construct	pFD288 (empty vector)	pBCX12	pBCX-A2	pBCX-C6 (with promoter)
Imipenem	0.032	0.032	2	0.25	32
Meropenem	0.032	0.032	0.25	0.125	32

a For the 5′ end of the constructs also see Figure S2.

PCR experiments were carried out as described previously^8^ and the primer sequences and PCR conditions are given in Table S1.

## Results and discussion

*B. xylanisolvens* 14880 was found to have the following MICs: imipenem, 128 mg/L; meropenem, >32 mg/L; ertapenem, >32 mg/L; doripenem, >32 mg/L; and imipenem/EDTA, 1 mg/L (the latter suggesting an MBL phenotype). Carrying out *cfiA* PCR on *B. xylanisolvens* 14880 yielded a negative result, but it produced 185 U/mg specific imipenemase activity. To investigate this carbapenem resistance mechanism, WGS and bioinformatic analyses were performed and antimicrobial susceptibilities were determined using Etest methodology (Table S2). The full genomic sequence obtained for *B. xylanisolvens* 14880 was 6.01 Mbp and it was resistant to only β-lactams (Table 2). Antibiotic resistance genes were detected using ResFinder and thus some known antibiotic resistance genes were revealed (Table S2). An ORF was also detected that belonged to the class B1 MBL group and had 37.3% identity and 82.3% similarity to CfiA (see Table 2 and Figure S1) at the protein level. The genomic sequence also revealed an IS*1380*-like IS element in its upstream region. Using 5′-RACE, we were able to determine the transcriptional start site 160 nt upstream of its start codon (Figure S2). Its gene transfer with various upstream sequences by triparental mating to a carbapenem-susceptible host (*B. fragilis* 638R) demonstrated that it could confer carbapenem resistance and therefore it was termed *crxA* (carbapenem resistance protein of *B. xylanisolvens*). Notably, the construct harbouring the IS*1380*-like IS promoter was able to increase the imipenem/meropenem MIC for the susceptible host strain 1000-fold (Table 2).

Despite the low identity value between CrxA and CfiA (37.3%), we managed to locate within CrxA the amino acid residues that make up the active centre of CfiA^16^ (Figure S1) and CrxA also harboured a C-terminal tail of 101 amino acids (Figure S1) compared with CfiA, but the effect of all the main differences on the enzymatic activity (e.g. substrate specificity and kinetic parameters) needs to be clarified in a future study.

IslandViewer located several putative chromosomal elements/islands in the genome of *B. xylanisolvens* 14880 (Figure S3), of which one is for *crxA* (Figure S4). In the *crxA* ‘element’ and in its vicinity there are regulatory genes, IS elements (IS*1380* and I*S110*-like ISs) and addiction toxin genes (Fic, GNAT) too. These and especially the latter make it somewhat similar to the *cfiA* gene island, which also contains a suspected GNAT toxin gene.

We also wished to determine the prevalence of *crxA* among *Bacteroides* spp.; strains from two earlier studies were screened for it by RT–PCR. Of the three non-*fragilis Bacteroides* strains of the first study, the *B. xylanisolvens* 22/1 strain proved to be positive, while of the strains of the second study, three *B. xylanisolvens* strains were positive (Table 1). We designed primers to detect the IS upstream of *crxA* in the latter strains, but a real activating one was just found in *B. xylanisolvens* 14880 (Table 1). Since MALDI-TOF MS cannot differentiate *B. ovatus* and *B. xylanisolvens* well, we used *rpoB* sequencing to determine the exact species identifications of the *B. ovatus/xylanisolvens* strains from the above two studies (Table 1). All the strains containing *crxA* turned out to be *B. xylanisolvens*, so we concluded that *crxA* is specific for *B. xylanisolvens* and estimated its prevalence among clinical *B. xylanisolvens* isolates in Hungary at 16.7% (3/18 *crxA*-positive *B. xylanisolvens*; Table 1).

Previously, the main carbapenem-resistant *Bacteroides* isolates were *B. fragilis*. Studies examining the faecal microbiota detected a significant proportion of resistant *Bacteroides* strains. In a recent study in our laboratory using selective media for *Bacteroides* isolation with (4 mg/L) and without meropenem, we found that about 1% of the cultivable *Bacteroides* population is resistant to carbapenems.^17^ Hansen *et al*.^18^ also noticed that after carbapenem treatment the prevalence of carbapenem-resistant *Bacteroides* strains increased in the normal microbiota of the treated patients and the majority of these strains were *B. xylanisolvens*. Recently some studies reported and characterized a few non-*fragilis Bacteroides* species isolates as carbapenem resistant,^9,19^ but this study is the most complete in characterization of the resistance mechanism.

### Conclusions

Overall, we can state that *B. xylanisolvens*, a non-*fragilis Bacteroides* species, can also harbour a carbapenem resistance mechanism that is similar to the *B. fragilis cfiA* system (MBL, IS element activation, a GNAT toxin gene-containing genetic element and a similar prevalence of ‘silent’ and resistant cases). Hence, as demonstrated by our current case and measured prevalences, carbapenem-resistant *Bacteroides* strains other than *B. fragilis* should not be ignored in the clinics.

## Supplementary Material

dkac088_Supplementary_Data

## Supplementary data

Tables S1 and S2 and Figures S1 to S4 are available as Supplementary data at *JAC* Online.

## References

[1] Wexler HM . *Bacteroides:* the good, the bad, and the nitty-gritty. Clin Microbiol Rev 2007; 20: 593–621. 17934076 10.1128/CMR.00008-07 PMC2176045

[2] Sóki J . Extended role for insertion sequence elements in the antibiotic resistance of *Bacteroides*. World J Clin Infect Dis 2013; 3: 1–12.

[3] Jurėnas D , Garcia-Pino A, Van Melderen L. Novel toxins from type II toxin-antitoxin systems with acetyltransferase activity. Plasmid 2017; 93: 30–5. 28941941 10.1016/j.plasmid.2017.08.005

[4] García N , Gutiérrez G, Lorenzo M et al Gene context and DNA rearrangements in the carbapenemase locus of division II strains of *Bacteroides fragilis*. Antimicrob Agents Chemother 2009; 53: 2677–8. 19364865 10.1128/AAC.01514-08 PMC2687242

[5] Sóki J , Hedberg M, Patrick S et al Emergence and evolution of an international cluster of MDR *Bacteroides fragilis* isolates. J Antimicrob Chemother 2016; 71: 2441–8. 27246231 10.1093/jac/dkw175

[6] Snydman DR , Jacobus NV, McDermott LA et al National survey on the susceptibility of *Bacteroides fragilis* group: report and analysis of trends in the United States from 1997 to 2004. Antimicrob Agents Chemother 2007; 51: 1649–55. 17283189 10.1128/AAC.01435-06 PMC1855532

[7] Hurlbut S , Cuchural GJ, Tally FP. Imipenem resistance in *Bacteroides distasonis* mediated by a novel β-lactamase. Antimicrob Agents Chemother 1990; 34: 117–20. 2327745 10.1128/aac.34.1.117 PMC171531

[8] Sóki J , Edwards R, Urbán E et al Screening of isolates from faeces for carbapenem-resistant *Bacteroides* strains; existence of strains with novel types of resistance mechanisms. Int J Antimicrob Agents 2004; 24: 450–4. 15519476 10.1016/j.ijantimicag.2004.06.017

[9] Sadarangani SP , Cunningham SA, Jeraldo PR et al Metronidazole- and carbapenem-resistant *Bacteroides thetaiotaomicron* isolated in Rochester, Minnesota, in 2014. Antimicrob Agents Chemother 2015; 59: 4157–61. 25941219 10.1128/AAC.00677-15 PMC4468676

[10] Chassard C , Delmas E, Lawson PA et al *Bacteroides xylanisolvens* sp. nov., a xylan-degrading bacterium isolated from human faeces. Int J Syst Evol Microbiol 2008; 58: 1008–13. 18398210 10.1099/ijs.0.65504-0

[11] Pedersen RM , Marmolin ES, Justesen US. Species differentiation of *Bacteroides dorei* from *Bacteroides vulgatus* and *Bacteroides ovatus* from *Bacteroides xylanisolvens* - back to basics. Anaerobe 2013; 24: 1–3. 23994205 10.1016/j.anaerobe.2013.08.004

[12] Sárvári KP , Sóki J, Kristóf K et al A multicentre survey of the antibiotic susceptibility of clinical *Bacteroides* species from Hungary. Infect Dis (Lond) 2018; 50: 372–80. 29303023 10.1080/23744235.2017.1418530

[13] Bortolaia V , Kaas RS, Ruppe E et al ResFinder 4.0 for predictions of phenotypes from genotypes. J Antimicrob Chemother 2020; 75: 3491–500. 32780112 10.1093/jac/dkaa345 PMC7662176

[14] Bertelli C , Laird MR, Williams KP et al IslandViewer 4: expanded prediction of genomic islands for larger-scale datasets. Nucleic Acids Res 2017; 45: W30–5. 28472413 10.1093/nar/gkx343 PMC5570257

[15] Smith CJ , Rollins LA, Parker AC. Nucleotide sequence determination and genetic analysis of the *Bacteroides* plasmid, pBI143. Plasmid 1995; 34: 211–22. 8825374 10.1006/plas.1995.0007

[16] Concha NO , Rasmussen BA, Bush K et al Crystal structure of the wide-spectrum binuclear zinc β-lactamase from *Bacteroides fragilis*. Structure 1996; 4: 823–36. 8805566 10.1016/s0969-2126(96)00089-5

[17] Sóki J , Wybo I, Hajdú E et al A Europe-wide assessment of antibiotic resistance rates in *Bacteroides* and *Parabacteroides* isolates from intestinal microbiota of healthy subjects. Anaerobe 2020; 62: 102182. 32126280 10.1016/j.anaerobe.2020.102182

[18] Hansen KCM , Schwensen SAF, Henriksen DP et al Antimicrobial resistance in the *Bacteroides fragilis* group in faecal samples from patients receiving broad-spectrum antibiotics. Anaerobe 2017; 47: 79–85. 28445776 10.1016/j.anaerobe.2017.04.013

[19] Kaeuffer C , Ruge T, Diancourt L et al First case of bacteraemia due to carbapenem-resistant *Bacteroides faecis*. Antibiotics (Basel) 2021; 10: 319. 33808699 10.3390/antibiotics10030319 PMC8003481

