# Supplementary material for: A novel Bacteroides metallo-β-lactamase (MBL) and its gene (crxA) in Bacteroides xylanisolvens revealed by genomic sequencing and functional analysis
Source: J Antimicrob Chemother. 2022 Mar 17;77(6):1553–6. doi: 10.1093/jac/dkac088 (PMC9472255; doi:10.1093/jac/dkac088)
Supplement: dkac088_Supplementary_Data [file dkac088_supplementary_data.docx]

**Supplementary data**

**Table S1.** Primers and PCR conditions

| Primer | Sequence 5’-3’ | PCR type | Cycles | Ref. |
| --- | --- | --- | --- | --- |
| cfiA1 | TCCATGCTTTTCCCTGTCGCAGTTAT | endpoint | 94 °C 30s, 50 °C 1 min, 72 °C 1 min; 35x | ^15^ |
| cfiA2 | GGGCTATGGCTTTGAAGTGC |  |  |  |
| crxAF | ACCGTTGGCAAAAGTAAGTT | RT-PCR | 95 °C 15s, 55 °C 30s, 72 °C 30s; 35x | This study |
| crxAR | TTCACACGGGCAATTGTATT |  |  |  |
| IS.crxA_F | CATAAGCTCGGTTCTCTGTG | RT-PCR | 95 °C 15 s, 55 °C 30 sec, 72 °C 30s; 35x^2b^ | This study |
| IS.crxA_R | ACAAGAAGTTCCTGGGCTAC |  |  |  |
| crxAR- IS.crxA_F | See above | PCR-mapping | 94 °C 15s, 55 °C 30s, 72 °C, 3 min; 35x | This study |
| crxA_up1 | GTTCCGGTGGAGTGGTAAAA | endpoint | 94 °C 30s, 60 °C 1 min, 72 °C, 3 min; 35x | This study |
| crxA_up2 | TGCGCATAAATATCACACCAA |  |  |  |
| crxA-F2 ^a^ | AACCCGGATCCAGCATCAAATTGCTCCATAA | endpoint (for cloning) | 94 °C 30s, 60 °C 30s, 72 °C 3 min; 35x | This study |
| crxA-F1 ^a^ | ACCCGGATCCTGTCTTCTTTTCGCAACACT |  |  |  |
| crxA-Fx ^a^ | ACCCGGATCCGTGTTGCGGAATAAAGGTAA |  |  |  |
| crxA-R ^a^ | ACCCGGATCCGCTTCTTGCCCCATCAAATA |  |  |  |
| crxA-GSP1 | ACCTTGATAGTATGCGCATA | RT^b^ | n.a.^C^ | This study |
| crxA-GS32 | AATATCACACCAACATAACAAAAG | 5’-RACE^d^ | 55 °C 10x, 60 °C 25x^d^ |  |
| crxA-GSP3 | TATAATTAAAAAGAAGTCTCTTAATTTCAT |  | 55 °C; 35x^b^ |  |
| rpoB-O1 | CCGTGAACGTGCCGGATT | endpoint | 94 °C 15s, 55 °C 30s, 72 °C 1 min; 35x | This study |
| rpoB-O2 | TTACCAACTGACGTTCGATACCT |  |  |  |

^a^ Forward (F) and reverse (R) primers are shown (*Bam* HI recognition site used for cloning is underlined). ^b^ Reverse transcription ^c^ Not applicable ^d^ We used the cycling parameters given in the 5’-RACE Kit Manual with the indicated annealing temperatures and cycle numbers.

**Table S2.** MICs of *B. xylanisolvens* 14880 to various antibiotics and the corresponding resistance genes

| Antibiotic | AMP^a^ | AMC | Ptc | FOX | IP | IPI | MP/EP/DP | ERY | CLI | MOX | MTZ |
| --- | --- | --- | --- | --- | --- | --- | --- | --- | --- | --- | --- |
| MIC (mg/L) | **>256**^b^ | 1 | **32** | **>256** | **128** | 1 | **>32** | 4 | 1 | 0.5 | 0.064 |
| Resistance gene | - | | | *cfxA* | *crxA* | | | *mef*(A) | | - | - |
| Homology |  |  |  | 99.7% | (novel) | | | 95.1% | |  |  |
| Antibiotic | TET | | TGC | CHL | LNZ | Rif | Aminoglycosides | | Sulfonamide | |  |
| MIC (mg/L) | 4 | | 0.125 | 1 | 2 | 0.003 | - | | - | |  |
| Resistance gene | *tet*(Q1) | *tet*(Q2) | - | - | - |  | *aph*(3')-III | *ant*(6)-Ia | sul2 | |  |
| Homology | 99.8% | 100% |  |  |  |  | 100% | 100% | 100% | |  |

^a^ AMP – ampicillin, AMC – amoxicillin/cavulanate, Ptc – piperacillin/tazobactam, FOX – cefoxitin, IP – imipenem, IPI – imipenem/EDTA, MP/EP/DP – meropenem, ertapenem, doripenem, ERY – erythromycin, CLI – clindamycin, MOX – moxifloxacin, MTZ – metronidazole, TET – tetracycline, TGC – tigecycline, CHL – chloramphenicol, LNZ – linezolide, Rif – rifampicine. ^b^ Resistant values are shown in bold.

**Figure S1.** Alignment of the *crxA* and *cfiA* genes. Stars and dots denote identities and degrees of similarities of amino acids, respectively. Residues necessary for binding the Zn^2+^ ions in CfiA and the corresponding sites in CrxA are marked red.

*crxA* MKLRDFFLII-LLLCWCDIYAHTIKVSDKLNLIQLNENVYIHTE---------NDNNGIV 50

*cfiA* --MKTVFILISMLFPVAVMAQKSVKISDDISITQLSDKVYTYVSLAEIEGWGMVPSNGMI 58

:: .*::* :*: . : :::*:**.:.: **.::** :.. .**::

*crxA* YINGGKAVIVSTPENDEETNYLIDYIRNHLKSEIVACVVDRW**H**P**D**AMGGLNAIKKANIPS 110

*cfiA* VINNHQAALLDTPINDAQTEMLVNWVTDSLHAKVTTFIPN**H**W**H**G**D**CIGGLGYLQRKGVQS 118

**. :*.::.** ** :*: *:::: : *::::.: : ::** *.:***. ::: .: *

*crxA* YANRLTQVIAKERMLPIPENGFDITLELTVGKSKLICHYLGEA**H**TKDGIVVWLPNEKILF 170

*cfiA* YANQMTIDLAKEKGLPVPEHGFTDSLTVSLDGMPLQCYYLGGG**H**ATDNIVVWLPTENILF 178

***::* :***: **:**:** :* :::. * *:*** .*:.*.******.*:***

*crxA* GGNQVRAK--GWYGNIGDANLREWSNTIARVKDLYGDAKIVIPG**H**GHYGGNELLDYTINL 228

*cfiA* GG**C**MLKDNQATSIGNISDADVTAWPKTLDKVKAKFPSARYVVPG**H**GDYGGTELIEHTKQI 238

** :: : ***.**:: * :*: :** : .*: *:****.***.**:::* ::

*crxA* YRPTLWGKILKWNDVQVKPVFNNCGVIFELAESDSINEGKRFLKNATIYIQQKNKNRYLK 288

*cfiA* VNQYIE--------STSKP----------------------------------------- 249

. : **

*crxA* IQSPMIRHDNEESQVLSSDKGRLQIYNITMNELIEDLYYKQLYISLEEQSVDALIILKEA 348

*cfiA* ------------------------------------------------------------ 249

*crxA* IR 350

*cfiA* -- 249

**Figure S2.** C*rxA* constructs with various upstream regions and the position of its transcription initiation site. Underlined nucleotides are sequences in primers used to construct pBCX-C6, pBCX-A2 and pBCX12, respectively. ^ denotes the transcription initiation site, determined by 5’-RACE, and < depicts the start of the IS*1380*-like IS. Capital bold letters denote the determined promoter sequence. The ATG shown in bold represents the start codon of the *crxA* gene.

^

agcatcaaattgctccataatagaaaaaattcctccaaaaggag**tg**agtttctcagattttat**t**tg**ta**tc**tttgC**catgtc

atattagagttttgcttgtcttcttttcgcaacactaaggtaagtgaaaattctgacatggcaaaatcctgggcaactttt

tgttgctcaggcacttataaataatgttaaactatagtgttgcggaattaagg**<**taatctaaaattgagatagtaga**ATG**a

**Figure S3.** The genome and genomic islands of *B. xylanisolvens* 14880.


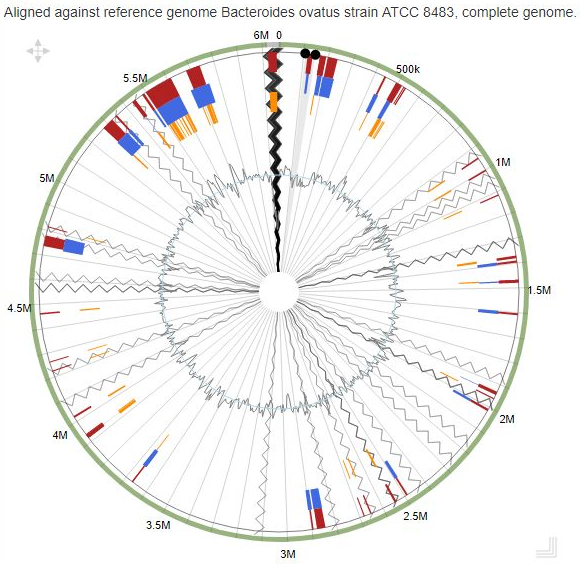


*crxA*

*cfxA*

*tet(Q1) tet(Q2)*

**Figure S4.** The genomic island of the *crxA* gene. The blue box denotes the ORFs placed into one genomic island by Islandviewer (marked as the *crxA* island on Figure S2).

DUT93_21660 GyrI-like domain-containing protein

DUT93_21655 PLP-dependent aminotransferase family protein

DUT93_21650 PLP-dependent aminotransferase family protein

DUT93_21645 Hypothetical protein

DUT93_21640 Hypothetical protein

DUT93_21635 Fic family protein

DUT93_21630 MATE family efflux transporter

DUT93_21625 Alpha/beta hydrolase

*crxA* Subclass B1 metallo-beta-lactamase

DUT93_21615 IS*1380* family transposase

DUT93_21610 Cupin domain-containing protein

DUT93_21605 Hypothetical protein

DUT93_21600 GNAT family N-acetyltransferase

DUT93_21595 Antibiotic acetyltransferase

DUT93_21590 IS110 family transposase

DUT93_21585 Hypothetical protein

DUT93_21580 Lrp/AsnC family transcriptional regulator
